# Could Neutrophil CD64 Expression Be Used as a Diagnostic Parameter of Bacteremia in Patients with Febrile Neutropenia?

**DOI:** 10.4274/tjh.2016.0123

**Published:** 2017-06-01

**Authors:** Nur Efe İris, Taner Yıldırmak, Habip Gedik, Funda Şimşek, Demet Aydın, Naciye Demirel, Osman Yokuş

**Affiliations:** 1 İstanbul Bilim University Faculty of Medicine, Department of Infectious Diseases and Clinical Microbiology, İstanbul, Turkey; 2 Okmeydanı Training and Research Hospital, Clinic of Infectious Diseases and Clinical Microbiology, İstanbul, Turkey; 3 Okmeydanı Training and Research Hospital, Clinic of Hematology, İstanbul, Turkey

**Keywords:** Neutrophil, CD64, Bacteremia, Febril neutropenia, Diagnostic parameter

## Abstract

**Objective:**

The aim of this study is to investigate if neutrophil CD64 expression in febrile neutropenia patients could be used as an early indicator of bacteremia.

**Materials and Methods:**

All consecutive patients older than 18 years of age who had developed febrile neutropenia episodes due to hematological malignancies were included in the study. Those patients who had significant growth in their blood cultures constituted the case group, while those who had febrile neutropenia without any growth in their cultures and who did not have any documented infections formed the control group. Blood culture bottles were incubated in the Bact ALERT 3D system (bioMerieux, France), identification and susceptibility testing were performed using an automated broth microdilution method (VITEK 2, bioMerieux), and CD64 expression analysis was performed by the flow cytometry method. C-reactive protein (CRP) was measured by turbidimetric methods (Biosystems, Spain) and erythrocyte sedimentation rate (ESR) was measured by the Wintrobe method.

**Results:**

In total, we prospectively evaluated 31 febrile episodes. The case group consisted of 17 patients while the control group included 14 patients. CD64 was found on neutrophils of the case group patients with a mean count of 8006 molecules/cell and of control group with a mean count of 2786 molecules/cell. CD64 levels of the case group were significantly higher than those of the control group (p=0.005). In the differentiation of the case group from the control group, a 2500 cut-off value for CD64 had significant [AUC=0.792 (0.619-0.965)] predictive value (p=0.001). In the prediction of patients with a 2500 cut-off value for CD64, sensitivity was 94.1%, positive predictive value was 76.2%, specificity was 64.3%, and negative predictive value was 90.0%. CRP levels and ESR values did not differ significantly between the groups (p=0.005).

**Conclusion:**

Neutrophil CD64 expression could be a good predictor as an immune parameter with high sensitivity and a negative predictive value for bacteremia in febrile neutropenic patients.

## Abstract (tr)

**Amaç:**

Çalışmanın amacı febril nötropenik hastalarda nötrofil CD64 ekspresyonunun bakteriyemi tanısında erken bir tanımlayıcı olarak kullanılıp kulanılamayacağını araştırmaktır.

**Gereç ve Yöntemler:**

Hematolojik malignite tanısıyla izlenip febril nötropeni atağı geçiren, 18 yaşından büyük olgular çalışmaya alındı. Olgulardan kan kültürlerinde anlamlı üreme saptanan grup olgu grubunu, febril nötropenik olup kültürlerinde üreme olmayan ve dökümante edilen klinik enfeksiyon bulgusu da olmayan olgular da kontrol grubunu oluşturdu. Kan kültürleri Bact ALERT 3D sisteminde inkübe edildi (bioMerieux, Fransa), identifikasyon ve antibiyotik duyarlılık testi otomatize broth mikrodilüsyon yöntemi (VİTEK 2) ile çalışıldı. CD64 ekspresyon analizi de flow sitometri yöntemi ile çalışıldı. C-reaktif protein (CRP) turbidimetrik yöntemle ölçüldü (Biosystems, İspanya), eritrosit sedimantasyon hızı da (ESH) Wintrobe yöntemi ile çalışıldı.

**Bulgular:**

Prospektif olarak 31 febril nötropenik atağı değerlendirildi. Olgu grubu 17 hastadan, kontrol grubu 14 hastadan oluşuyordu. Olgu grubundaki hastalarda nötrofiller üzerindeki CD64 molekülü sayısı ortalama 8006 molekül/hücre ve kontrol grubunda ise ortalama 2786 molekül/hücre idi. Olgu grubundaki CD64 düzeyleri kontrol grubunun değerlerinden anlamlı olarak yüksek bulundu (p=0,005). Olgu grubunun kontrol grubundan ayrımında CD64 için 2500 cut off değeri anlamlı bulundu [AUC=0,792 (0,619-0,965)] prediktif değer (p=0,001). Hastaların tahmininde CD64 için 2500 lük cut off değerinin duyarlılığı %94,1; pozitif prediktif değeri %76,2, duyarlılığı %64,3, negatif prediktif değeri %90 olarak hesaplandı. Gruplar arasında CRP düzeyleri ve ESH değerleri arasında anlamlı bir farklılık saptanmadı (p=0,005).

**Sonuç:**

Nötrofil CD64 ekspresyonu febril nötropenik hastalarda bakteriyeminin tanısında yüksek duyarlılıklı ve negatif prediktif değere sahip iyi bir parametre olabilir.

## INTRODUCTION

Patients with neutropenia are very prone to bacterial infections, and especially in severely neutropenic patients, it is difficult to diagnose bacterial infection due to the lack of local signs of infection. Fever may be the only sign. For patients with febrile neutropenia, bacteremia is a very severe clinical presentation because these patients can easily progress to sepsis and septic shock. Positive blood culture is the gold standard for bacteremia but there are some difficulties. Difficulty in exclusion of infection is harmful in febrile neutropenia patients with suspected sepsis, as continuation of broad-spectrum antibiotics for presumptive bacterial infection frequently leads to unnecessary treatment and the possibility of multiresistant organisms. It brings toxicity, allergic reactions, and increased cost, too [1,2,3]. Thus, we need an improved diagnostic test for detecting bacterial infection and bacteremia in febrile neutropenic patients in the early period.

Acute phase reactants like C-reactive protein (CRP) have been used as indicators of bacterial infections since the 1970s. However, CRP has some limitations; for example, it is elevated in noninfectious processes. Procalcitonin (PCT) is a more reliable test for sepsis, especially as it has a very good negative predictive value for sepsis. PCT is more widely used in the diagnosis of severe sepsis and bacterial infections. It is also used in monitoring the success of antimicrobial treatment. PCT is secreted upon exposure to endotoxin. However, it might be increased in noninfectious conditions, too, like severe congestive heart failure and acute pancreatitis [2,3,4,5,6,7]. Many investigators have used various hematologic and biochemical markers and cytokines such as tumor necrosis factor-α, interleukin (IL)-1β, soluble IL-1ra, IL-2 receptor, IL-6, IL-8, IL-10, and markers of complement-activation for this purpose. Another approach in the early diagnosis of sepsis is neutrophil CD64 expression [1,8,9,10,11,12,13,14].

Neutrophils are very important cells for antibacterial immunity. Their basic function is phagocytosis. The surface receptors of neutrophils recognize bacterial antigens and this interaction activates the neutrophils to phagocytosis. Phagocytosis is facilitated by various receptors for immunoglobulin-G (IgG) and neutrophils can express three classes of IgG receptors. Fc gamma receptor I (Fc gamma RI) is recognized by the monoclonal antibody CD64 [12]. CD64, a high-affinity immunoglobulin Fc gamma RI, is constitutively expressed on monocytes but upregulated during acute-phase reactions on polymorphonuclear neutrophils (PMNs). Measured on PMNs, CD64 expression is a sensitive biomarker for bacterial infection [2,12,15,16,17].

In febrile neutropenic patients, can we use neutrophil CD64 expression as a rapid diagnostic tool as an indicator of bacteremia? The aim of our study was to answer this question. Our goal was to determine if neutrophil CD64 expression has a significant correlation with microbiologically documented bacterial infections and if neutrophil CD64 expression can predict the results of a patient’s blood culture.

## MATERIALS AND METHODS

### Subjects

This single-center prospective study was carried out at the Okmeydanı Training and Research Hospital in İstanbul, Turkey. All consecutive patients between February 2013 and July 2013, who were older than 18 years of age and who had developed febrile neutropenic episodes during chemotherapy due to hematological cancers in the hematology department, were included in the study. Febrile neutropenia was defined as an oral temperature of >38.3 °C or two consecutive readings of >38 °C for 2 h and an absolute neutrophil count of <0.5x109/L or a count expected to fall below 0.5x109/L. We collected data including sex, age, comorbidity, and suspected origin of fever, and we collected clinical and laboratory data including body temperature, blood pressure, complete blood count, liver enzymes, creatinine, prothrombin time, CRP, chest radiographs, and urine, sputum, and blood cultures. Thirty-one episodes of febrile neutropenia in patients undergoing chemotherapy were selected independently of the type of underlying hematological disease.

**I. Case group:** This group included patients with a confirmed diagnosis of sepsis, which was defined by positive blood culture results.

**II. Control group:** This group included patients without sepsis, with suspected infection but negative blood culture results.

Single episodes of febrile neutropenia were considered; thus, the same patient may have been included more than once. Patients undergoing chemotherapy and with fever but with absolute neutrophil counts of >1.0x10^9^/L were excluded from the study.

We performed blood cultures for each patient in the first episode of the febrile neutropenic period and before starting antibiotic therapy. We placed 10 mL of blood in aerobic and anaerobic blood culture bottles and incubated them in the Bact ALERT 3D system (bioMerieux, France). Identification and susceptibility testing were performed using an automated broth microdilution method (VITEK 2, bioMerieux).

We measured neutrophil CD64 expression by flow cytometry following the manufacturer’s instructions. The BD QuantiBRITE CD64/CD45 Phycoerythrin Florescence Quantitation Kit (Becton Dickinson, USA) was used. Quantitative flow cytometric analysis of the ethylenediaminetetraacetic acid specimens was performed with the FACSCalibur machine (Becton Dickinson). The cytometer was routinely optimized using CaliBRITE beads (Becton Dickinson). Before each analysis, QuantiBRITE polyethylene (PE) beads conjugated with four predefined levels of PE molecules were used to construct a standard linear regression curve. The amount of PE molecules bound per cell standard for the absolute number of fluorochrome antibody binding sites per cell was calculated. In principle, each QuantiBRITE grade antibody molecule is conjugated to one PE fluorochrome molecule. The instrument measures the CD64 antigen, which is one of three Fc receptors for immunoglobulins, including human Fc gamma RII (CD32) and human Fc gamma RIII (CD16) antigens, present on the surface of leukocytes.

QuantiBRITE kits contain calibration beads for the fluorescent labels. CD64 was measured using the BD QuantiBRITE CD64 PE/CD45 PerCP assay (Becton Dickinson). The assay was carried out essentially as described in the product instructions. Samples consisting of 50 µL of blood were stained in polystyrene tubes of 12x75 mm by adding 20 µL of staining reagent and incubating at room temperature (20-25 °C) for 30 min in the dark. Then 1.0 mL of FACS lysing solution was added to each tube, and the tubes were vortexed at low speed for approximately 1 or 2 s and incubated at room temperature for approximately 5 min in the dark. The level of CD64 expression, reported as antibodies bound per cell, was determined as described in the product instructions.

Serum concentrations of CRP were measured by turbidimetric methods (Biosystems, Spain). Erythrocyte sedimentation rate (ESR) was measured by the Wintrobe method.

### Statistical Analysis

Our primary aim was the comparison of two groups for their neutrophil CD64 expressions. We determined the standard effect size as 1.05 for the two groups before the study. It was decided to include 14 patients for each group as the minimum, with 80% power and 5% confidence interval. Three more patients were kept as a reserve for the case group. In the descriptive statistics of the data, mean, standard deviation, median, lowest value, highest value, and percentage were used. The distribution of the variables was measured with the Kolmogorov-Smirnov test. The Mann-Whitney U test was used for analyzing the quantitative data. Effect level and cut-off values were assessed with ROC curves. For correspondence analysis, the kappa test was used. SPSS 22.0 was used for analyses.

## RESULTS

Thirty-one episodes of febrile neutropenia were analyzed; analyzed episodes that corresponded to patients with two or more episodes of neutropenia were considered as separate events and were included as such in the analysis. Among the 31 included events, 17 episodes involved positive blood culture results and 14 involved negative results. Regarding the blood cultures, coagulase-negative methicillin-resistant *Staphylococcus* was isolated in eight episodes (all patients had clinical signs and this organism was isolated in three blood culture bottles), *Escherichia coli* in five (ESBL-positive *E. coli* in two), *Pseudomonas aeruginosa* in two, *Klebsiella pneumoniae* in one, and *Kluyvera ascorbata* in one.

**Figure 1:**
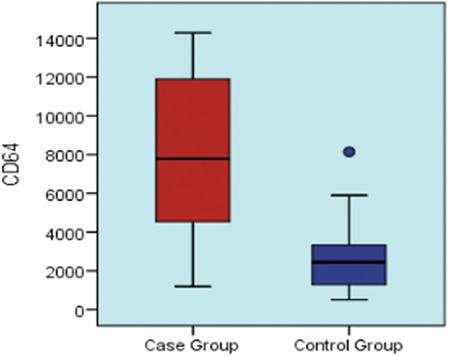
Box plot graph shows the comparison between CD64 expression (molecules/cell) in the case group (mean ± standard deviation: 8006±4243; median: 7786) and the control group (mean ± standard deviation: 2876±2149; median: 2452) (p=0.001).

CD64 was found on neutrophils of case group patients at a mean count of 8006 and median count of 7786 molecules/cell and on the neutrophils of the control group at a mean count of 2876 and median count of 2452 molecules/cell (Figure 1).

CD64 was found on neutrophils of gram-negative bacteremia patients at a mean count of 8562 molecules/cell and on neutrophils of gram-positive bacteremia patients at a mean count of 7380 molecules/cell.

**Figure 2:**
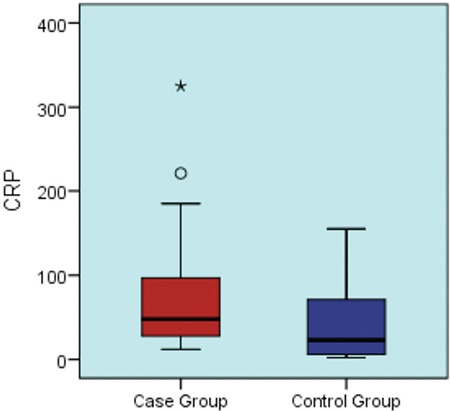
Box plot graph shows the comparison between C-reactive protein (mg/L) in the case group (mean ± standard deviation: 82±86.4; median: 48) and the control group (mean ± standard deviation: 43.4±47.5; median: 23) (p=0.084).

**Figure 3:**
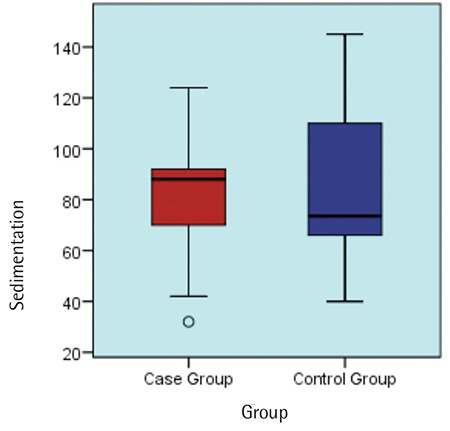
Box plot graph shows the comparison between erythrocyte sedimentation rate (mm/h) in the case group (mean ± standard deviation: 79.6 ±26.3; median: 88) and the control group (mean ± standard deviation: 82.7±27.5; median: 74) (p=0.827).

The mean CRP level was 82 mg/L in the positive culture group and it was 43 mg/L in the negative culture group (Figure 2). The mean ESR level was 79 mm/h in the positive culture group and 82 mm/h in the negative culture group (Figure 3). CRP and ESR levels did not differ significantly among the groups (p=0.084 and p=0.005; Table 1).

**Figure 4:**
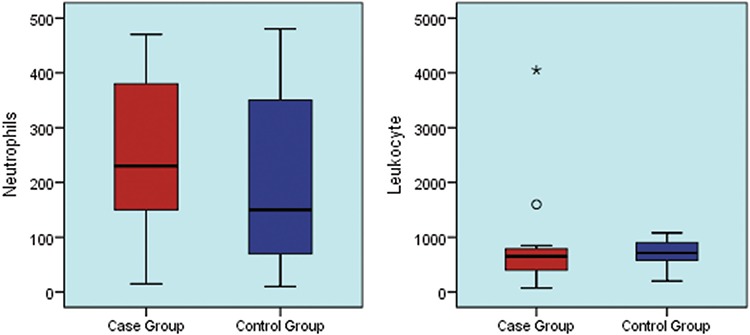
Box plot graph shows the comparison between neutrophils and leukocytes (/µL) in the case group (mean ± standard deviation: 246±144 and median: 230; mean ± standard deviation: 795±906 and median: 650) and the control group (mean ± standard deviation: 218±167 and median: 150; mean ± standard deviation: 706±246 and median: 715) (p=0.827).

The mean total leukocyte count was 0.618x10^3^/µL and the mean neutrophil count was 0.245x10^3^/µL in the group with positive blood culture (case group), while mean total leukocyte count was 0.706x10^3^/µL and mean neutrophil count was 0.124x10^3^/µL in the group with negative blood culture (control group) (Figure 4).

**Figure 5:**
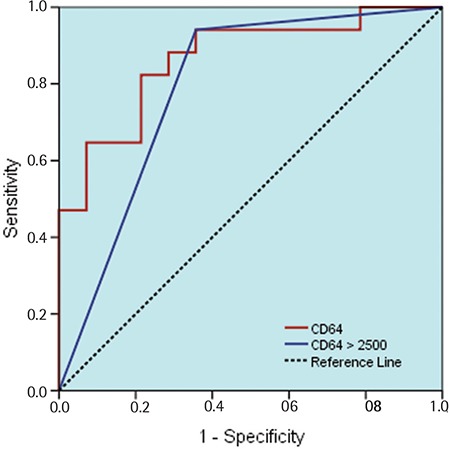
Sensitivity and specificity of CD64 levels.

CD64 levels of the bacteremia group were significantly higher than those of the control group (p=0.005). In the distinction of the study group from the control group, CD64 levels had a significant [AUC=0.866 (0.737-0.994)] predictive value (p=0.001; Table 2). In the differentiation of the case group from the control group, a cut-off value of 2500 molecules/cells for CD64 had a significant [AUC=0.792 (0.619-0.965)] predictive value (p=0.001). In the prediction of patients with a 2500 cut-off value for CD64, sensitivity was 94.1%, positive predictive value was 76.2%, specificity was 64.3%, and negative predictive value was 90.0% (Figure 5). CD64 expression in cases of gram-negative bacteremia was higher than in cases of gram-positive bacteremia, but this was a statistically nonsignificant difference (p=0.564).

## DISCUSSION

If bacteremia is not quickly treated in febrile neutropenia patients, it becomes a significant risk factor for septic shock. That is why it is crucial to get positive blood culture results as early as possible. The activation of neutrophils is evidence for the presence of bacteria in the circulation. For this reason, being able to show the activation of neutrophils on the first day of the febrile neutropenia episode is of vital importance and can serve as an alarm much earlier than the culture results. Showing the expression of CD64 on the surface with flow cytometry is the earliest indicator of neutrophil activation [1,2,12,13,14,15,16,17].

Is it possible to identify febrile neutropenia in patients with bacteremia before their microbiological culture results are obtained, and is there a test that would help us predict that the cause of fever in febrile neutropenic patients is an infection? Could CD64, an indicator of neutrophil activation, be used for this purpose? In this study, which was performed with the aim of answering these questions, the results that we obtained favor our hypotheses.

CD64 levels of the bacteremia group were significantly higher than those of the control group and, in the distinction of the groups, CD64 levels had a significant predictive value. In the prediction of patients with a 2500 cut-off value for CD64 (molecules/cells), sensitivity was 94.1%, positive predictive value was 76.2%, specificity was 64.3%, and negative predictive value was 90.0%.

Sepsis causes significant morbidity and mortality among febrile neutropenic patients. Up to 35% of sepsis patients have no identified microbiological agent and therefore diagnosis and empiric therapy have to be based on other parameters [18,19,20]. The lack of specific criteria makes the search for sensitive and specific diagnostic biomarkers crucial. As measurements of markers like CRP and PCT can be obtained much earlier than culture results, they are very useful in the early diagnosis of sepsis. However, their sensitivities are low. CRP and ESR are markers that indicate inflammation, and malignancy ranks first among the conditions that result in their elevation. To this end, in patients with malignancies, like the febrile neutropenia patients in our study, their benefits in the differential diagnosis of an infection would be limited [5,21,22,23,24].

In the literature, in a study that was very similar to ours, it was investigated whether CD64 expression was a predictor for positive blood cultures in febrile neutropenic children or not and furthermore CRP and ESR were compared. Median CD64 indices were found to be similar both in the negative and the positive blood culture groups. A correlation could not be identified between CD64 index and positive blood culture. Furthermore, for CRP levels, there was no statistically significant difference between the study groups with positive and negative blood cultures and the control group [25].

In a meta-analysis evaluating neutrophil CD64 expression as a marker for bacterial infection, patients from all age groups were considered and CD64 expression on neutrophils was identified as a useful cell-based diagnostic tool for the diagnosis of bacterial infections. The sensitivity of neutrophil CD64 expression was found as 79% and its specificity as 91%. Based on the evaluation made in this meta-analysis, 13 prospective studies were analyzed and among them only those studies enrolling adult patients, those in which CD64 measurements were performed with flow cytometry, and those which confirmed the presence of an infection with blood culture positivity were shown to prove the sensitivity and specificity of CD64 [26].

In a study investigating the benefit of using neutrophil CD64 expression to discriminate between exacerbation of the disease and the addition of an infection to the clinical presentation in inflammatory autoimmune diseases, neutrophil CD64 expression was reported to have a sensitivity of 94.4% and a specificity of 88.9% [27]. Furthermore, neutrophil CD64 density was higher in gram-negative bacterial infections despite not reaching levels of statistical significance and this was explained by the activation of neutrophils by different bacterial products through different pathways. In our study, we found that patients who had growth of gram-negative bacteria also had higher levels of CD64 expression despite the fact that this was not statistically significant. If the results of further studies with higher numbers of patients support our results, it could provide us with an extremely useful diagnostic opportunity that might guide us in the choice of antibiotherapy.

According to our study, neutrophil CD64 expression is a good diagnostic marker for bacteremia in febrile neutropenic patients. However, an infection marker should be biochemically stable because it might be necessary to keep blood samples stored, but flow cytometric analysis is not available for this situation. The advantages of CRP when compared with CD64 are rapid quantitation and easy handling. The quantitative flow cytometric analysis of CD64 was applied in this study. CD64 could be developed into a routine clinical test with high reproducibility and easy handling so that it can be used easily as a biomarker of bacteremia for febrile neutropenia patients.

In the literature there are some studies that demonstrated that CD64, when measured as an index, has high sensitivity and specificity for infection and monitoring sepsis [28,29,30]. The CD64 index was measured using a Leuko64kit (Trillium Diagnostics, Bangor, ME, USA) and a BD FACSCalibur running QuantiCALC software (Verity Software House, Topsham, ME, USA) in these studies. Many modern hematology analyzers contain flow cytometers and are capable of doing CD4 subsetting, and incorporation of CD64 index testing into these platforms will further simplify this test [28].

The main limitation of our study is the number of patients included. We recommend performing more studies with a higher number of patients. In this way it will be possible to obtain more powerful data on using CD64 and predicting bacteremia.

## CONCLUSION

It is obvious that more studies focused on neutrophil CD64 expression for the diagnosis of bacteremia in febrile neutropenic patients are needed. According to our results, neutrophil CD64 expression is a good diagnostic tool for the diagnosis of bacteremia in febrile neutropenia patients, and CD64 is superior to CRP and ESR.

## Figures and Tables

**Table 1:**
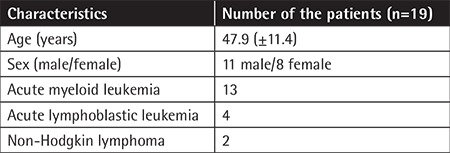
Characteristics of the study group.

**Table 2:**
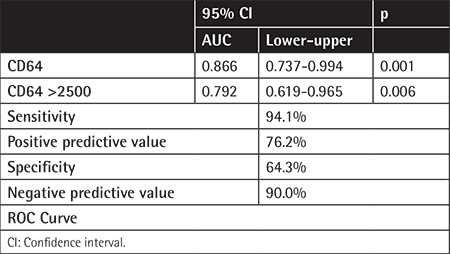
Sensitivity, specificity, and predictive values of CD64.
